# A dedicated automatic dilutor for the automation of α_2_ macroglobulin kinetic studies

**DOI:** 10.1155/S1463924683000097

**Published:** 1983

**Authors:** C. Riley, J. D. McK. Watson, J. Morgon, B. F. Rocks, M. S. Sheya, P. R. Oldfield, C. Ewen

**Affiliations:** 1 School of Engineering and Applied Sciences University of Sussex Brighton BN1 9QT UK; 2 Biochemistry Department Royal Sussex County Hospital Brighton BN2 5BE UK; 3 Department of Electrical Engineering University of Dar es Salaam Tanzania; 4 School of Molecular Sciences University of Sussex Brighton BN1 9QT UK


Journal of Automatic Chemistry, Volume 5, Number 1 (January–March 1983), pages 27–31

### A dedicated automatic dilutor for the automation of $\alpha_2$ macroglobulin kinetic studies

**C. Riley, J. D. McK. Watson, J. Morgon**
*School of Engineering and Applied Sciences, University of Sussex, Brighton BN1 9QT, UK*

**B. F. Rocks**
*Biochemistry Department, Royal Sussex County Hospital, Brighton BN2 5BE, UK*

**M. S. Sheya**
*Department of Electrical Engineering, University of Dar es Salaam, Tanzania*

**P. R. Oldfield and C. Ewen**
*School of Molecular Sciences, University of Sussex, Brighton BN1 9QT, UK*

In 1979, evidence was produced by Topping and associates [1, 2 and 3] that any one of at least seven different types of $\alpha_2$ macroglobulin ($\alpha_2$M) is to be found circulating in human blood. Serum samples from healthy individuals showed a reproducible distribution amongst the seven categories, which differed markedly from the distribution obtained from a group of patients suffering from a variety of pulmonary diseases. Subsequent studies (to be published) on a wider spectrum of diseases confirmed these findings and strengthened the view that the different categories have an important bearing on disease processes and probably have diagnostic and prognostic significance. The studies undertaken so far have involved the ability of $\alpha_2$M to bind trypsin. Additional, unpublished, evidence suggests that studies involving the supplementary use of other proteolytic enzymes are likely to add considerably to the clinical usefulness of the studies.

These seven categories of $\alpha_2$M were identified by measuring the ability of a range of dilutions of serum to inhibit the activity of bovine trypsin. A series of dilutions of serum in Tris [tris (hydroxy methyl) methylamine] buffer was prepared and trypsin added to each dilution. After incubation for 10 min or more, the hydrolytic activity of the trypsin was measured by adding an aliquot of each dilution in turn to a substrate containing N-Benzoyl-L-arginine ethyl ester (BAEE) and measuring the rate of absorbance change at 253 nm. The rates obtained are believed to be a measure of the rate of partition of trypsin between circulating proteinase inhibitors. Plots of the rates obtained at various dilutions have a consistent form in which four rates can be identified; variations in this partition profile provide the correlation with disease states.

This technique is time-consuming: preparation of the set of dilutions and measurement of the reaction rates takes a minimum of 2 h of concentrated labour, followed by tedious mathematical treatment. This work has tremendous promise but, for the studies to reach their full potential, large numbers of analyses need to be carried out and so some form of mechanization was urgently needed. As a first approach it seemed reasonable to separate the dilution step from the kinetic measurements and mechanize each independently. The present paper describes the construction of a dilutor, which automatically prepares 25 dilutions from a single sample of serum. A stopped-flow/flow-injection system was proposed for the kinetic measurements; this has been constructed and is at present under evaluation.

### Design of the dilutor

The range of dilutions required is shown in table 1. The spacing of the individual dilutions has been varied in order to concentrate the maximum number of points at the inflections of the plot. This rules out a simple mechanical approach, so it was decided to operate the device under computer control.

The measuring component consists of two syringes (figure 1), the pistons of which are propelled by screw-threads driven by

*Table 1. Volume of serum and buffer dispensed.*

| Tube number | Serum volume (μl) | Buffer volume (μl) |
|---|---|---|
| 0 | 0 | 400 |
| 1 | 5 | 395 |
| 2 | 10 | 390 |
| 3 | 15 | 385 |
| 4 | 20 | 380 |
| 5 | 25 | 375 |
| 6 | 30 | 370 |
| 7 | 35 | 365 |
| 8 | 40 | 360 |
| 9 | 45 | 355 |
| 10 | 50 | 350 |
| 11 | 55 | 345 |
| 12 | 60 | 340 |
| 13 | 80 | 320 |
| 14 | 100 | 300 |
| 15 | 120 | 280 |
| 16 | 140 | 260 |
| 17 | 160 | 240 |
| 18 | 180 | 220 |
| 19 | 200 | 200 |
| 20 | 220 | 180 |
| 21 | 260 | 140 |
| 22 | 300 | 100 |
| 23 | 340 | 60 |
| 24 | 380 | 20 |
| Totals 25 tubes | 2·87 ml serum | 7·13 ml buffer |

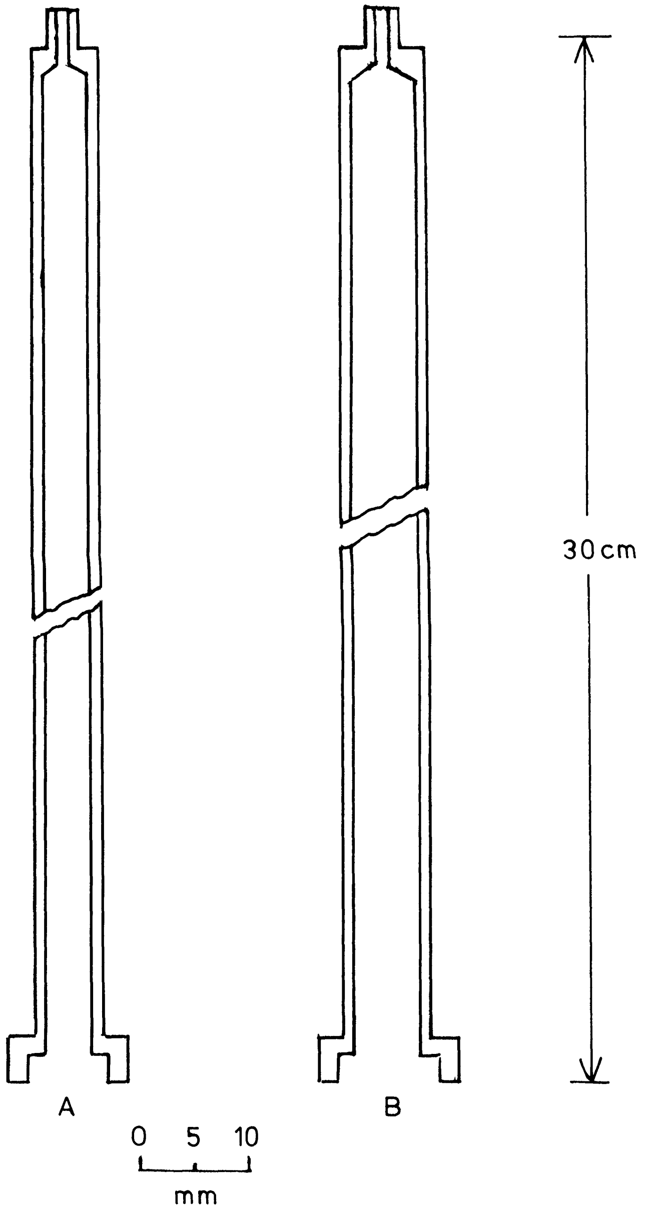

*Figure 1. 'Syringes': A = serum syringe and B = buffer syringe.*

stepping-motors. The barrels of the syringes are 300 mm long, and the pistons are 4 mm and 6 mm diameter for the serum and diluent respectively. The diameter of the syringes was kept as small as possible in relation to the length, this was to minimize the inevitable errors in linear movement. The barrels were constructed from commercially available poly-methyl methacrylate (Perspex) tubing and were approximately 0·5 mm larger in diameter than their pistons. The upper ends were closed off with machined plugs provided with a 4 mm diameter spigot with a 1 mm diameter exit hole. The bases of the syringes are thick discs which were machined from sheet Perspex, with recesses to accept nitrile rubber 'O' rings. These were of British Standard sizes BS 007 and 010 respectively. The recesses for the 'O' rings were machined according to BS 1806. The plugs and bases were cemented in place with Tensol cement No. 6 (ICI Plastics Division, Welwyn Garden City, Hertfordshire, UK).

The two pistons were made from different materials: the serum piston is only in contact with distilled water and is of 316 stainless-steel. The diluent syringe, however, is filled with Tris buffer (at pH 8·14) which contains chloride ions; so its piston was made of Incoloy 825 (a nickel, chromium, molybdenum and iron alloy) which is more resistant to chloride ions than are stainless-steels (Incoloy 825 is supplied by Henry Wiggin & Co., Bakers Road, Uxbridge, Middlesex, UK).

As can be seen from table 1, the 25 different dilutions of serum in buffer give a final volume of 400 $\mu$l in each case. The serum volume increases in increments of 5 $\mu$l, or multiples of 5 $\mu$l, and the buffer solution decreases in corresponding decrements. The smaller (serum) syringe has a 4 mm diameter piston driven by a 5 mm metric thread of 0·8 mm pitch. The stroke length for 5 $\mu$l is 0·3979 mm (0·4 mm). So 180° rotation of the motor for each 5 $\mu$l is needed. The motors used (Impex I.D. 27, Dennard Rotadrive, Mill Lane, Alton, Hampshire, UK) have $7\frac{1}{2}$° step angles so the requirement is 24 steps per 5 $\mu$l; the actual stroke volume for 24 steps is 5·029 $\mu$l. The larger (buffer) syringe has a 6 mm diameter piston driven by a 4 B.A. thread of 0·660 mm pitch. The stroke length for 5 $\mu$l is 0·1768 mm or 12·858 steps of $7\frac{1}{2}$°, 13 steps give a stroke volume of 5·054 $\mu$l. Thus the theoretical dilution error is acceptably small. The general arrangement of the syringes and their drive mechanisms is illustrated in figure 2. The overall length of the whole assembly is over 1 m, so to save space it is mounted vertically at the end of the bench. It is an advantage to arrange the syringes with their tips upwards— air is then easily displaced.

The diluted mixture is discharged into autoanalyser-type polystyrene cups. The 25 cups are mounted at 20 mm centres in a stand made of folded stainless-steel sheet, they are arranged to slide past the delivery point of the serum and diluent. The stand travels between flanged nylon guide discs which are free to revolve (figure 3). Attached to the base of the stand is a rack with

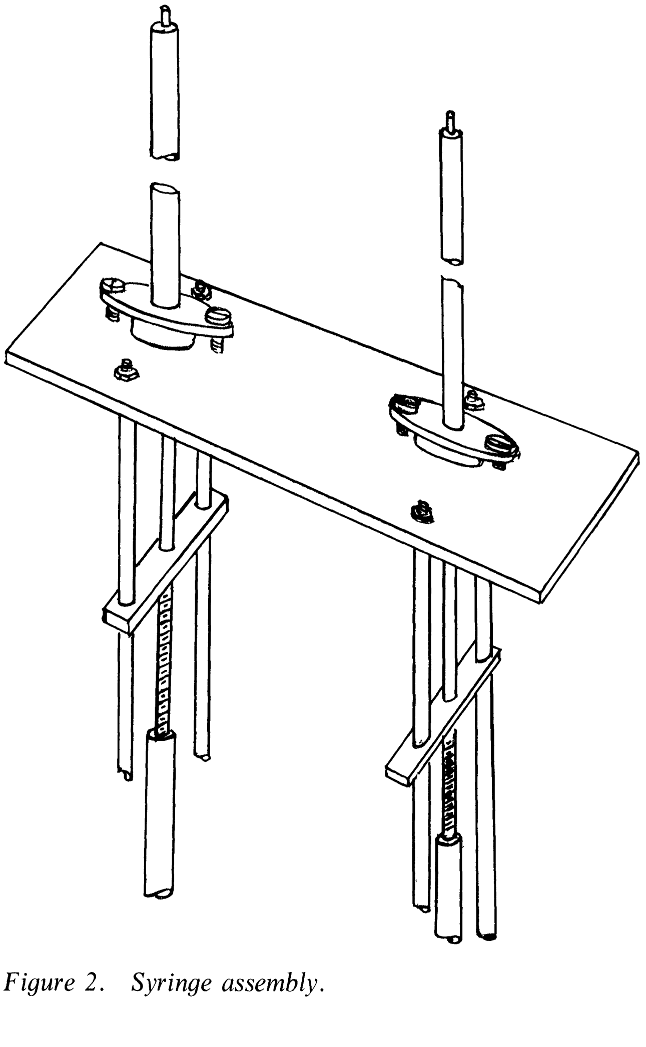

*Figure 2. Syringe assembly.*

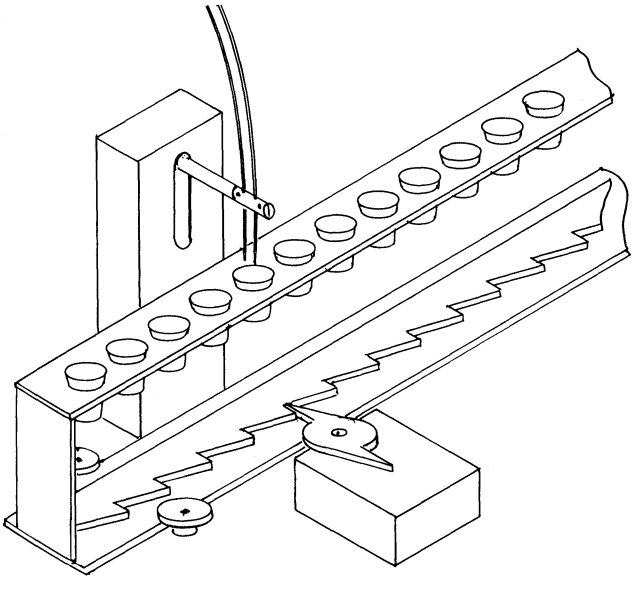

*Figure 3. Sample cup stand and dipping mechanism.*

skewed teeth of 20 mm pitch, which is driven by a pinion with two opposed teeth. The pinion is mounted on the output shaft of a geared synchronous instrument motor of 30 r.p.m. output (supplied by McLennon Servo Supplies Ltd, Camberley, Surrey, UK). Also mounted on the gearbox shaft are a pair of magnets of a Hall-effect switch, which signals the control electronics to interrupt the power-supply each time the pinion makes a half revolution. Working in conjunction with this is the dipping mechanism (see figure 3). This consists of a crank-operated slide driven by another geared instrument motor and also has a Hall-effect device to arrest it at the top of its stroke. A bracket mounted on the slide holds the two delivery tubes 3 cm from their tapered tips, which are dipped below the surface of the diluted mixture after each delivery in order to remove the last droplets. The dipping mechanism is mounted in such a position that the tips of the probes lie vertically above each cup presented to them.

The probes are of PTFE electrical sleeving (HW 19, Polypenco, from G. H. Bloore Ltd, Solent Road, Havant, Hampshire, UK) of 1·0 mm bore, drawn out to narrow tips. The diluent probe is connected directly to its syringe; the serum probe, on the other hand, is continuous with a 3 m coil of tubing which is connected to its respective syringe. In use, only distilled water enters the serum syringe—thus minimizing carry-over between samples. The whole assembly is mounted on the bench as close as possible to the upper ends of the syringes.

### *Controller electronics and software*

Factors such as ease of use, low cost and flexibility were considered paramount in the design of the controller subsystem. It was felt that the unit should not be unnecessarily sophisticated, and that it should 'stand alone' without the need for a VDU, or other console device. Hence, beyond the capability of automatically co-ordinating the set of dilutions defined in table 1, the controller was provided with only limited facilities for manual control and alteration of dilution pattern. In addition to a small custom-designed microcomputer embodying a Z80 microprocessor, the controller comprises two stepper-motor power-supply and driver subsystems and a mains-driven synchronous motor interface. Physical dimensions of the unit are 213 × 133 × 240 mm.

Front-panel controls include eight dedicated keys to initiate the dilution sequence, fill and empty syringes continuously or by steps, reset the microcomputer and select the dilution pattern. These are defined by parameter tables stored in EPROM; although only one is currently employed, provision was made for one of up to 10 to be selected. Further flexibility in pattern adjustment accrues from the reprogrammability of the parameter table EPROM. A numeric display shows the pattern selected and LED lamps indicate machine status. The microcomputer is a minimal realization (figure 4) with partially decoded memory space and memory-mapped input/output (I/O). Two 2 k × 8 EPROMs provide program and regime parameter stores respectively, whilst 1 k × 8 of static RAM is used as scratch-pad memory. A feature of the stepper-motor power-supply and driver subsystem is a software-controlled 'shut-down' facility. This allows stepper-motors not in use to be powered down, thereby minimizing unnecessary heat dissipation in the controller. The controller, which is interfaced to the stepper-motor driven syringe pumps and magazine positioning and probe dipper mechanisms, receives positional information from Hall-effect limit switches on these units. Inputs are provided to signal syringes full or empty, magazine at 'home' (initial) position and magazine indexed. Corresponding outputs control the stepper and synchronous motors of the pump and magazine subsystems.

Software was written in PL/8080, a language similar to ALGOL, which encourages structured programming practice and facilitates a programmer throughput higher than that of assembly language. The modular approach adopted comprises three major components: a command recognizer, a command implementer and a body of utility and driver routines. Arranged hierarchically, the program has at its highest level the command recognizer, followed by the command implementer which forms a multiply nested subroutine structure. At the lowest level, utility routines perform all I/O management and system-timing functions, such as the generation of stepper-motor signal pulses.

Inputs from the control keys are interpreted by a simple 'parser', as entries may be single or multiple depending on mode of operation. When the system is under manual control, a multiple key sequence defines subject, direction and movement type in order. If, for example, it is desired to fill the serum syringe, the required key sequence would be; 'SERUM', 'FILL', 'STROKE'. 'SEQUENCE ACTIVATE' and 'REGIME SELECT' are single key commands. Incorrect entry sequences and pump/magazine initialization errors are visually flagged and further operations are locked out until the system is reset.

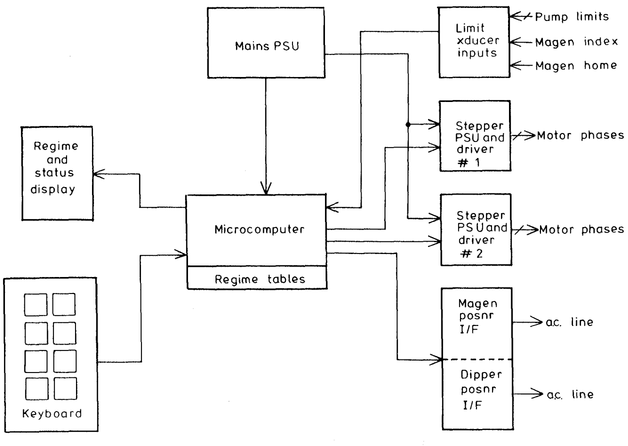

*Figure 4. Controller block diagram.*

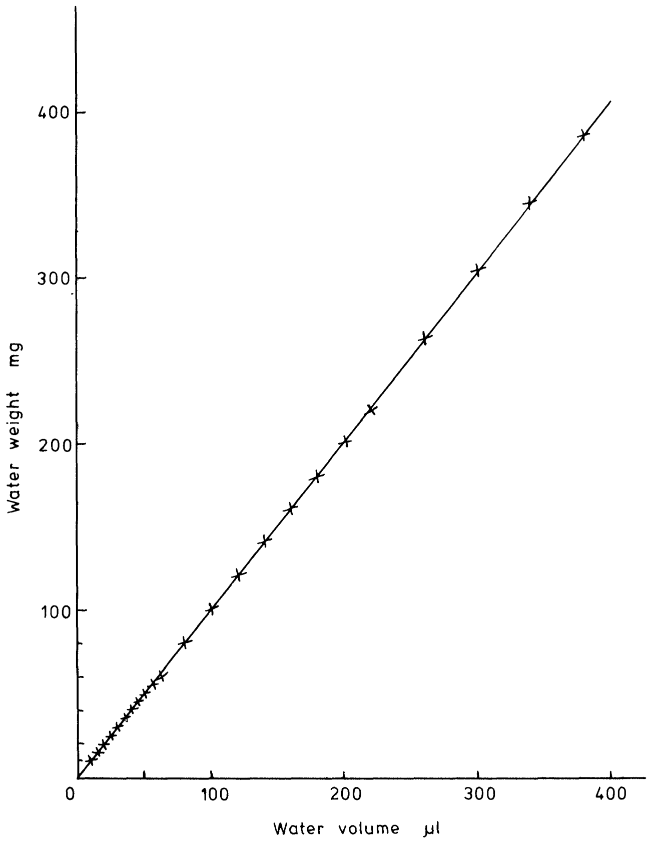

*Figure 5. Weight of water dispensed (serum syringe).*

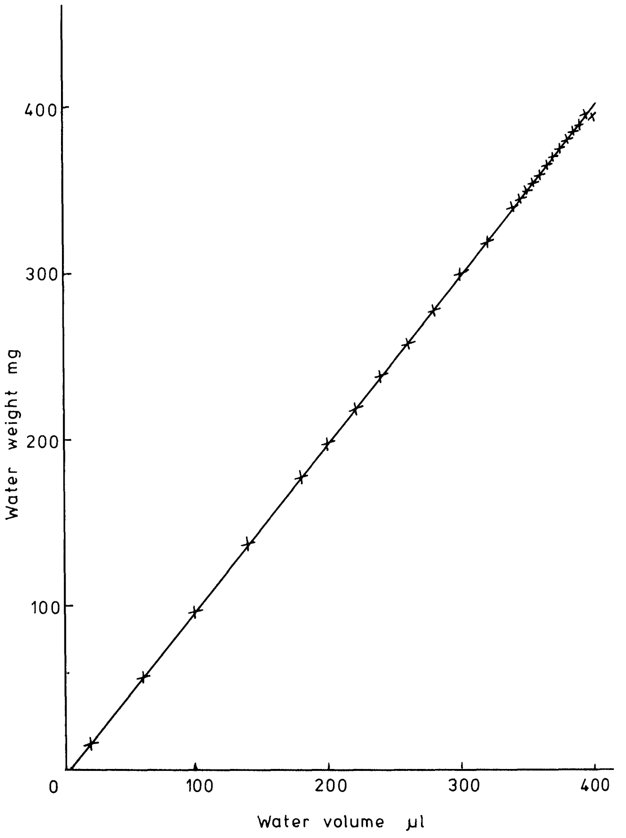

*Figure 6. Weight of water dispensed (buffer syringe).*

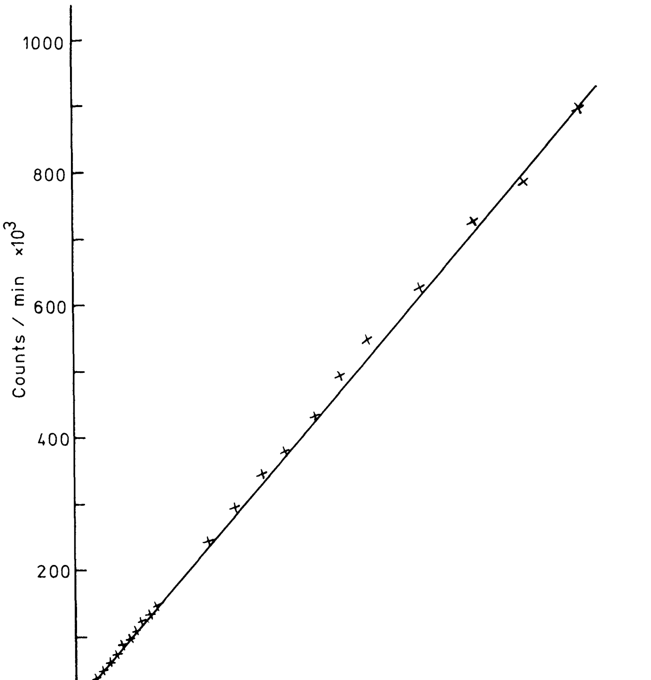

*Figure 7. Delivery and dilution of radio-labelled serum.*

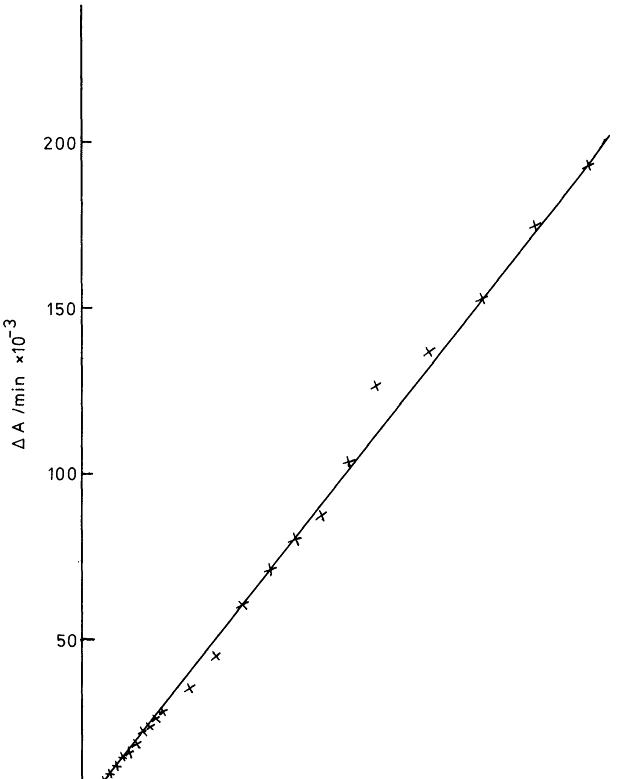

*Figure 8. Kinetic activity of different volumes of a trypsin/serum mixture.*

### Operation of the system

As a preliminary, the serum syringe is completely filled with distilled water; it is usually necessary to eject this and refill to displace trapped air. The water is again ejected, the piston withdrawn slightly to create a small air-bubble and serum drawn in, almost filling the reservoir coil of tubing. The diluent syringe is filled with buffer, if necessary ejecting it once to clear trapped air. The 25 cups are mounted in their stand and the mechanism started. When all 25 dilutions have been prepared, the cups are transferred for analysis. The reservoir coil is then detached from its syringe and flushed with a little water before aspirating the next sample of serum.

### Validation of the dilutor

Each syringe was tested independently by filling it with distilled water and discharging it into one of the polystyrene cups, capping and weighing it between each discharge. Figures 5 and 6 show plots of the incremental weights against volumes theoretically delivered, and, as can be seen, these are remarkably accurate. In addition, a sample of trypsin labelled with $^{125}I$ was mixed with serum and dispensed and diluted by the dilutor. After stirring, 0·25 ml aliquots were transferred into polystyrene tubes and counted in an NE 1600$\gamma$ scintillation counter (Nuclear Enterprises Ltd). Figure 7 shows counts/min plotted against the theoretical volume of serum delivered. To test for carry-over, water was dispensed from the serum syringe after the experiments with radio-labelled trypsin. The water was pooled and counted in the gamma counter. This was carried out twice and on neither occasion was the count above background.

As a final measure, a sample of fresh serum was mixed and incubated with a large excess of trypsin and diluted with buffer on the machine. The reaction kinetics of each dilution against BAEE was then measured spectrophotometrically. Figure 8 shows the plot of serum volume delivered against tryptic activity. Bearing in mind the vagaries of these particular reaction kinetics the results are quite satisfactory.

As mentioned earlier, the second phase of mechanization has involved the construction of a stopped-flow/flow-injection system. This is now under evaluation and will be the subject of a later paper.

### References

1. Topping, R. M. and Seilman, S., *Biochemical Journal*, **177** (1979), 493.
2. Topping, R. M. and Craven, A. H., *Biochemical Journal*, **177** (1979), 501.
3. Topping, R. M., Craven, A. H., Whiting, S., Rigden, B. G., Turner-Warwick, M. and Turton, C. W. G., *Clinical Science*, **60** (1981), 261.

### MEETING ANNOUNCEMENTS

### *Practical sampling systems for on-line process measurement*

Organized by Sira, and to be held on 18 May 1983 at the Meeting Rooms of the Zoological Society of London, Regent's Park, London NW1, the seminar is a follow-up to those held in 1981 and 1978 on the same topic. The objective is to provide an overall view of the use of sampling systems for on-line process measurement for liquids and gases, and also for slurries, pastes and solids, and to show through practical examples the methods which have been adopted in various industries.

The morning session will consist of four keynote lectures covering the use of sampling systems for liquids and gases; sampling of flue gases, sampling of slurries, pastes and solids; and sampling for environmental monitoring. The afternoon session will include presentations by speakers from a range of industries, and a group discussion period in which speakers will lead groups in the discussion of a variety of topics of interest to delegates.

The seminar is primarily intended for instrument engineers, plant laboratory analysts and process control engineers. Literature about commercially available equipment will be displayed at the seminar.

*Details from the Conference Unit, Sira Institute Ltd, South Hill, Chislehurst, Kent BR7 5EH, UK. Tel.: 01 467 2636.*

### *Computing in Clinical Laboratories*

This is the fourth international meeting on Computing in Clinical Laboratories and will be held in Breda, The Netherlands, from 24–26 August 1983. The programme is designed for clinical laboratory workers and computer scientists and is divided into three main sessions: Introduction of computer systems into pathology laboratories; Computer networks; and Computer-assisted interpretation of laboratory data.

*Further details from Mr R. C. J. Galle, Stichting Medische Laboratoria, Bergschot 69, 4817 PA Breda, The Netherlands.*

### *International Symposium on Electroanalysis in Biomedical, Environmental and Industrial Sciences*

Organized by the Electroanalytical Group and Western Region of the Royal Society of Chemistry, this Symposium will be held at UWIST in Cardiff from Tuesday 5 April to Friday 8 April 1983. The programme will be on aspects of electroanalysis relating to biomedical, environmental and industrial sciences. The methodology and applications of the various electroanalytical methods will be emphasized, and especially the use of amperometric and potentiometric membrane and membrane-clad electrodes, gas sensors, polarography. Development, operation and mechanisms will be included where these relate to electroanalyses in the theme areas, including enzymes and substrates.

Anyone wishing to read a paper should contact *Dr G. J. Moody* at the *Chemistry Department, UWIST, Cardiff CF1 3NU, UK.*

